# More evidence or stronger political will: exploring the feasibility of needle and syringe programs in Ukrainian prisons

**DOI:** 10.1186/s12954-020-00459-z

**Published:** 2021-01-19

**Authors:** Alexandra Dmitrieva, Vladimir Stepanov, Kateryna Svyrydova, Ievgeniia-Galyna Lukash, Svetlana Doltu, Mikhail Golichenko, Valeriy Kalivoshko, Evgeniy Khanyukov, Zhannat Kosmukhamedova, Oleh Torkunov, Oleksii Zagrebelnyi

**Affiliations:** 1 Support, Research and Development Center, Kyiv, Ukraine; 2 Doctoral School at National University of “Kyiv-Mohyla Academy” (NaUKMA), Kyiv, Ukraine; 3 Council for Preventing and Eliminating Discrimination and Ensuring Equality, Chisinau, Republic of Moldova; 4 Council for the Prevention of Torture (National Preventive Mechanism), Chisinau, Republic of Moldova; 5 HIV Legal Network, Toronto, Canada; 6 State Penitentiary Service, Kyiv, Ukraine; 7 Global Fund and PATH Projects in State Penitentiary Service, Kyiv, Ukraine; 8 Regional Programme Office for the Eastern Europe United Nations Office on Drugs and Crime, Kyiv, Ukraine; 9 Kyiv Regional State Administration, Kyiv, Ukraine; 10 FREEZONE, Kyiv, Ukraine

**Keywords:** Carceral collectivism, Injecting drug use, Post-Soviet prison, Soviet legacies

## Abstract

**Introduction:**

In 2007, the World Health Organization (WHO) recommended for prison authorities to introduce prison needle and syringe programs (PNSP) if they have any evidence that injecting drug use is taking place in prisons. This article presents descriptive evidence that injecting drug use takes place in Ukrainian prisons, it discusses how (denial of) access to injection equipment is regulated in the current system and what changes should be considered in order to implement PNSP.

**Background:**

Ukrainian prisons still live by the laws and policies adopted in the Soviet Union. Besides laws and regulations, these legacies are replicated through the organization and infrastructure of the prison’s physical space, and through “carceral collectivism” as a specific form of living and behaving. Inviolability of the prison order over time helps the prison staff to normalize and routinely rationalize punishment enforcement as a power “over” prisoners, but not a power “for” achieving a specific goal.

**Methods:**

The Participatory Action Research approach was used as a way of involving different actors in the study’s working group and research process. The data were gathered through 160 semi-structured interviews with prison health care workers, guards, people who inject drugs (PWID) who served one or several terms and other informants.

**Results:**

The “expertise” in drug use among prisoners demonstrated by prison staff tells us two things—they admit that injecting use takes place in prisons, and that the surveillance of prisoner behavior has been carried out constantly since the very beginning as a core function of control. The communal living conditions and prison collectivism may not only produce and reproduce a criminal subculture but, using the same mechanisms, produce and reproduce drug use in prison. The “political will” incorporated into prison laws and policies is essential for the revision of outdated legacies and making PNSP implementation feasible.

**Conclusion:**

PNSP implementation is not just a question of having evidence of injecting drug use in the hands of prison authorities. For PNSP to be feasible in the prison environment, there is a need for specific changes to transition from one historical period and political leadership to another. And, thus, to make PNSP work requires making power work for change, and not just for reproducing the power itself.

## Introduction

The discussion of needle and syringe programs’ relevance in the prison environment was initiated by national and international health agencies not later than in 1987 [1]. This reflects the changes in global agendas regarding the health of PWID who are overrepresented in prisons and prisoners’ health issues in general. These agendas sequentially position PNSP as a health care service equivalent to that, which is provided to PWID in the community [2–4], as an essential harm reduction measure reducing the risks of HIV and Hepatitis C/B [5–11], and as a human rights and drug policy priority [12–15].

In 2007, the WHO recommended for prison authorities to introduce PNSP if they have any evidence that injecting drug use is taking place in prisons [11]. The exact number of PWID held in custody in Ukraine is unknown. Although it is known that around 14% of all prisoners are subjected to imprisonment for drug-related offenses every year, excluding crimes committed to finance their drug use [16, 17]. For example in 2018, of those convicted for drug crimes, 8,513 people (84%), were convicted of crimes for drug possession for personal use and, of those, 6,482 (76%) were convicted for possession of drugs in miniscule amounts that ranged from 0.005 to 1 g of heroin [18]. Despite a well-studied link between injecting drug use, imprisonment, and increased risk of HIV spread [19–26], implementing PNSP remains a rare harm reduction measure worldwide. According to the latest report on the Global State of Harm Reduction, only 10 countries were implementing PNSP in at least one prison in 2018, among those countries were Armenia, Canada, Germany, Kyrgyzstan, Luxembourg, North Macedonia, Moldova, Spain, Switzerland and Tajikistan [27]. Ukraine does not implement PNSP in prisons even though it has the second-largest HIV/AIDS epidemic in Eastern Europe and Central Asia after Russia, and that the epidemic is concentrated especially among PWID [28].

In February 2020, the UN Committee on Economic, Social and Cultural Rights recommended for Ukraine to “expand harm reduction programs, particularly in prisons” [29]. As a party to the International Covenant on Economic, Social, and Cultural Rights, Ukraine should respect the recommendations of the Committee. According to the Law of Ukraine On International Treaties of Ukraine [30], international treaties should have priority in case of conflict with domestic laws and must be implemented in Ukraine in good faith. According to article 27 of the Vienna Convention on the Law of Treaties [31], Ukraine may not invoke the provisions of its internal law as justification for its failure to perform a treaty, such as the International Covenant. This means that if Ukraine chooses to ignore the recommendations given by the Committee, it should provide very strong reasons for this. The reasons must be significantly justified if such health care interventions as needle and syringe programs are available in the community and funded by the Government because according to the United Nations Standard Minimum Rules for the Treatment of Prisoners, prisoners should enjoy the same standards of health care that are available in the community [32]. Also, the reasons for the refusal to adopt PNSP must be very strong if the Committee’s recommendation is supported by the national epidemiological data and scientific research.

This article presents descriptive evidence that injecting drug use takes place in Ukrainian prisons based on interviews with prison staff members. Furthermore, the article aims to discuss how the prison order was constructed in the Soviet past and is represented in the current system, including through ways the (denial of) access to injection equipment is regulated. Finally, we suggest what changes should be considered to implement PNSP in the researched field.

## Background

### Soviet legacies

According to previous studies, Ukrainian prisons still live by the laws and policies adopted in the Soviet Union [33, 34]. Much of that system has been copied into the Pre-Trial Detention Centers Rules and Prison Facilities Rules (further—the Rules) and into some other basic documents, for example, regulating the provision of health care in prisons.

The Rules are by-laws that have undergone several stages of review after the Soviet Union collapsed in 1991. However, despite a large number of formal amendments and changes in terminology, they essentially preserve the basic paradigms and provisions of the Soviet internal prison regulations developed by the Ministry of Internal Affairs of the USSR [33]. The Rules contain a large number of unjustified prohibitions which lead to corruption, lack of legitimacy of prohibitions and thus non-compliance, and selective application of the routine regulations. The general paradigm of the Rules claims to follow the goals of discipline and security—as being primary while humane standards such as providing adequate health care to prisoners always play a secondary role [35, 36]. Inviolability of the Rules over time not only sustains the old system, but helps the prison staff to normalize and routinely rationalize punishment enforcement [37], its supreme and even “sacred” role in the prison as a power “over” (le pouvoir «sur»), but not a power “for” (le pouvoir «pour») [38, 39]. Oleinik wrote that “the prison guards exercise it less to achieve a specific goal (be it "rehabilitation," "retribution" or "isolation") than to ensure the maintenance of order and the subject's obedience. The concept of power "over" emphasizes the obedience of one person to the will of the other” [39]. Consequently, it can be suggested then, that the prison order not only influences the lives of prisoners but of staff members as well [40], since the Rules manifest an absence of distinction between the private and public life of prisoners through legitimizing power of prison staff to constantly survey and control both as their main work duty [38].

The relevant prison legislation treats needles and syringes as medical equipment that can only be prescribed by health care professionals in case of a prisoner’s need “to get treatment necessary to maintain the vital functions of the body through injections in the presence of an appropriate medical prescription” [41, 42] (like in case of insulin treatment for people with diabetes). In all other cases, needles and syringes are strictly prohibited in pre-trial detention centers and prison facilities. At the same time, only a specially trained health care professional in the field of “narcology,” a drug dependence treatment field established in the early Soviet Union [43] can register a fact that a person uses/injects drugs and diagnose drug dependence, and thus following this logic, (s)he could prescribe needles and syringes as medical equipment.

Although this logic sounds inapplicable in the case of drug use, the existing legal frames do not consider “drug use” separately from “drug dependence” and its treatment. According to Raikhel, the approach postulated through the field of narcology was “a behavioral, rather than a pharmacological treatment and has generally depended upon, and helped to reinforce, clinical encounters premised on a tight hierarchical physician–patient relationship” [44]. In the prison environment, this approach is exacerbated by the “double loyalty” of prison staff and thus a duty [35, 45] – to treat and punish simultaneously. At the same time, a need to be registered as a “drug user” or “drug dependent” refers to “narcouchet”—a practice established in 1988, through the joint Order issued by the USSR Ministry of Internal Affairs and the Ministry of Health, which declared a joint responsibility for the registration and monitoring of drug users. The order required all individuals using psychoactive substances to be forced to undergo treatment, placed on a register and have regular examinations by “a narcologist.” That order was a starting point for the doctors at narcological facilities to cooperate and share information with law enforcement agencies on a routine, legitimized basis [46].

Unlike the solitary confinement system, the post-Soviet penitentiary system enforces prisoner cohabitation in large barracks, where dozens or even hundreds of people can end up living. In pre-trial detention centers, people are held in cells shared with many others as well. As other researchers denote, the “carceral collectivism” as a Soviet legacy is a cornerstone which has to be dealt with to make prison reform in post-Soviet space effective [47–49]. This form of collectivism is inspired by Anton Makarenko’s collective pedagogy which postulated that “more active participation of self-management collectives in the rehabilitation of inmates will result in the increased role of mutual dependency and responsibility, making it necessary for members of the collective to evaluate and influence each other” [50]. At the same time, it is a form of collective identity aimed at extensive mutual surveillance developed under the auspices of the prison administration and that of the informal governance of the criminal elite [47, 48]. The prison staff in the post-Soviet context can also be represented as a form of a collective whose work, attitudes, and beliefs are organized by way of the Rules. According to Makarenko, the life of any community is aimed at fostering labor discipline, each member of the community makes a commitment and takes responsibility, learns to immediately and without complaint follow work orders that he receives from superiors and must comply with the established "standards" of the work, which are considered as "minimal" [51].

Thus, the prison system is one of the vivid examples of how the Soviet legacies established in the far past continue to be reproduced in post-Soviet Ukraine [52]. On the one hand, they are replicated through legal documents and regulations, on the other—through the organization and infrastructure of the prison’s physical space, and finally—Soviet legacies are reproduced through “carceral collectivism” as a specific form of living, working and behaving in post-Soviet prison settings.

### Present day trends

At the end of 2015, the Ministry of Justice of Ukraine (MOJ) declared 2016 as the Year of prison reform [53]. Soon after that, the MOJ abolished the State Penitentiary Service (SPS) as a central executive body. Another change was the establishment of the State Institution "Health Center of the State Penitentiary Service of Ukraine" on November 1, 2017 [54]. However, an integration of the penitentiary healthcare system into the public healthcare system of the Ministry of Health of Ukraine did not happen. The interaction between the medical units and the administration of the penitentiary facility remains uncertain and leads to various, sometimes distorted, practices of cooperation and coordination [54].

A new trend that appeared after 2015 is associated with the way that prison drug use problems have started to be discussed within the SPS authorities. One of the illustrations of this change were the Facebook posts of the head of the SPS security department about the number of prison guards that have been detained as a result of their attempts to smuggle drugs to prisoners. According to his posts, 43 guards were detained in 2016, 46 in 2017, and 59 in 2018. As a result of long-lasting advocacy work in the previous period, at the beginning of 2020, the first pilot Opioid Agonist Therapy program (OAT) was approved by the Order No. 4092/5 of MOJ dated December 26, 2018, finally started being implemented in one Ukrainian prison—a male prison for persons who were sentenced to imprisonment more than once. This article is based on the findings of the study “Exploring the feasibility of PNSP in pre-trial detention centers and prison facilities in Ukraine,” conducted during October 2018—September 2019. To the best of our knowledge, this was the first qualitative study aimed at exploring the feasibility of PNSP in Ukraine. It could be suggested that feasibility of conducting a study of this kind was also a question of the SPS leadership’s interest and support.

In autumn 2019, when the new president of Ukraine was elected and the new cabinet of ministers was appointed, almost all previous SPS’s leaders finished their work in the prison system. Those leaders came to power as a result of the Revolution of Dignity that happened in Ukraine in 2014. The new minister of justice has already recommended himself as a change-maker through implementing an experimental program of fee-based cells in pre-trial detention centers to establish a budget for developing better conditions in the ordinary cells. However, it is not clear for now if the new leaders of the MOJ and SPS have PNSP as a priority for further development. The significant changes coming as a result of new political leadership and their agenda are known as one of the possible reasons for suspending PNSP in other countries [55–57]. A similar situation happened in Ukraine in 2005, after the Orange Revolution, the SPS announcing the start of a pilot PNSP in two prisons [58]. However, those pilot programs have never been implemented due to political changes in the country that happened shortly afterwards [52, 55, 59].

## Methods

The general approach to conducting this study was based on the principles of Participatory Action Research (PAR) [60]. The PAR implementation model [61, 62] in the process of research practice consists of four main dimensions: (a) context, (b) group dynamics, (c) interventions, (d) results. Context is a set of social determinants (for example, culture, environment, politics, which structure the perception and understanding of any process by researchers and research participants). Group dynamics are individual and group aspects of the interaction between researchers and research participants that are formed during the course of the research. Specificities of context and group dynamics affect the design and implementation of interventions and outcomes (for example, a change in practice or the way hierarchies are built).

McIntyre noted [60] that PAR is an ongoing cyclical process of interaction through which a “collective” of outsiders and insiders in relation to the topic/community/system under study collaborates in defining priorities, needs, designing interventions, and interpreting results. The processes used in PAR research are guided by principles, not methodological rules, and are flexible, adapted to the context and relationships between “insiders” and “outsiders.” In particular, PAR was used as a way of involving different actors in the study’s working group and research process designed to motivate changes at the level of the prison system. The actors were represented by international and national stakeholders; prison authorities; prison healthcare workers; and representatives of the network of non-governmental organizations advocating for the rights of current and former prisoners as well as supporting them.

Before the fieldwork began in February 2019, information webinars developed together by the working group members were held for the prison staff, including health care workers. The webinars were held on the territory of the SPS Administration in Kyiv. The webinars were moderated by the Deputy Head of the SPS Administration to ensure the observance of the rights of convicts and persons taken into custody (OT). A member of the Equality Council and the Council for the Prevention of Torture in Moldova (SD) spoke about the practice of implementing harm reduction programs in Moldovan prisons. Issues regarding the harm reduction approach as part of a prisoner’s right to health care were highlighted by a senior analyst of the Canadian HIV/AIDS Legal Network (MG). The audience of the webinars consisted of employees of penitentiary institutions, including medical workers. A total of three webinars were held and were attended by 75 employees of the pre-detention centers and prison facilities.

Support of the working group in organizing the fieldwork in pre-detention and prison facilities was crucial. Both, the SPS and the MOJ authorities played a crucial role in making this study possible in the context of extreme bureaucracy and a certain level of resistance at the ground level.

All working group members participated in the series of meetings to discuss the preliminary and final results. Their engagement in discussion of the results led to developing the presented article, co-authored by the researchers and all participants of the working group.

### Data gathering

The data were gathered through semi-structured interviews that were conducted according to pre-approved guides prepared for each type of informant. To identify the initial topics for developing interview guides, a literature review (n = 82) of existing experience of PNSP implementation worldwide was conducted. The following topics were covered:

- prevalence of injecting drug use before and during imprisonment,
- organization and functioning of interventions/services in the field of HIV/AIDS prevention and treatment within the prison system,
- organization and functioning of “hidden” interventions, i.e., informal measures aimed at reducing harm from drug use and maintaining health, both on the part of prisoners themselves and on the part of prison staff,
- opportunities for harm reduction interventions’ institutionalization within the prison system.

To understand the prevalence of injecting drug use at one or another prison facility, every informant was asked three consecutive questions:

1. In your opinion, how many of the inmates had been injecting drugs before being imprisoned?
2. How many of them do you think continue to inject drugs while being imprisoned?
3. Are there those who had not injected drugs before their sentence, yet started doing it in prison?

Interviews with all types of informants ended with a block of questions to discuss informants’ perceptions of existing models of PNSP implementation in specific countries which were presented by the interviewers, and how they could work/not work in the Ukrainian context. On average, the interviews lasted between 1 and 1.5 h.

During the whole research period, all 4 researchers (AD, VS, KS, IL) kept an online field diary, which, in addition to reflecting on the interviews, reflected the study coordination, data gathering process, interaction with all study participants, including prison administration and recruiters, the emotional atmosphere in which the interviews were conducted, and difficulties that were encountered in the field. Thus, a written field diary of more than 100 pages of printed text served as an additional tool for us to gather data and at the same time as a form of self-help and group supervision.

### Study locations

The study locations (penal facilities and pre-trial detention centers) were selected based on their geographical variability, institutional type variability, and reported HIV prevalence rate. A total number of 20 pre-trial detention centers (SIZOs^1^) and prison facilities (colonies) were included: male facilities (n = 11), female facilities (n = 5), and pre-trial detention centers (n = 4) in 7 largest regions out of the total 27 (including those where the largest cities of Ukraine are located: Kyiv, Dnipro, Odesa, Lviv). This means that in the remaining 20 regions the situation can vary in comparison to the data presented in this article. For example, the prison facilities sample did not include maximum-security regime prison facilities. Additional research is needed to fill this gap. Among the 16 prison facilities in the study (excluding 4 pre-trial detention centers), half were for those serving sentences for the first time and another half for those who had been imprisoned before. ﻿The sample included facilities of medium (n = 8) and minimum-security regimes (n = 6), and so-called “correctional centers” (CCs) (n = 2) where the regime of liberty restriction instead of liberty deprivation is in operation.

### Study participants

Informants were recruited using purposeful sampling with a maximum variation strategy in terms of informants’ professional and social positions within the prison system. The way a high heterogeneity of the sample was saturated is indicated in Table 1. The overall number of interviews conducted in this study is 160.

**Table 1:** Types and numbers of informants

Type of Informant	Number of informants
National and international stakeholders	5
SPS national and regional heads	5
Medical workers	24
Prison guards	39
Probation service workers	7
Social workers with experience of work with people who are serving their sentence/or have been released from prison	40
PWID who have been released from prison after their first sentence (0–12 months after release)	20
PWID who have served more one term and have recently been released from prison (0–12 months after release)	20
Total	**160**

Eligible PWID were recruited in Kyiv, Odesa, Dnipro, and Lviv. In each city, an NGO supporting current and former prisoners assisted with their recruitment. Among the interviewed PWID (n = 40) in this study, only 14 were sentenced solely for the possession and trafficking of drugs. The remaining 26 were sentenced both for possession and trafficking of drugs as well as for property crimes committed mostly to finance their drug use or drug dependence (theft, robbery, burglary). The ratio of men and women in the study was not the same in terms of interviews with former prisoners. Of the 40 former prisoners interviewed, only 6 women took part in the study. This gap was partially filled by data obtained from interviews with women-social workers (n = 10), who had experienced imprisonment. This limitation indicates difficulties in recruiting women who inject drugs and had an experience of serving a sentence in prison. At the same time, this indicates a high need for additional research oriented specifically at women prisoners, given that women prisoners and their (post) prison experience overall are much less studied worldwide and in Ukraine in particular.

All the interviews with prison staff members were conducted at their workplace. In particular cases it was impossible to ensure confidentiality and one-on-one conditions. Among the interviewed prison staff (n = 63), only three persons in one of the female prison facilities completely refused to discuss either prison drug injecting within the prison or PNSP implementation models. The interviews with healthcare workers from Kyiv and Kyiv region prisons (n = 6) were conducted in a rather difficult environment—in one room, in which three interviewers, located at the maximum possible distance from each other, were interviewing two health workers each at the same time. This method of interviewing can affect the quality of the data gathered, as informants may influence each other during the interview process, thereby distorting the individual opinion of a particular person. Thus, this can also be considered as a limitation of the study.

### Data analysis

The interviews were audio-recorded and then transcribed verbatim to be coded with the help of MAXQDA software. Two researchers (AD, VS) were involved in the coding process. The thematic analysis of the data was conducted to create a coding system that would meet the research objectives. After double coding, the researchers discussed the differences using a constant comparison method [63]. After that, the rest of the transcripts were coded; quotes with similar codes were sorted and categories were created, which were then combined into broader topics. The field diary data were also coded and helped to contextualize what informants had said in the interviews with what had happened during the interviews and field period in general.

## Results

### In their own voices: how prison staff perceive prevalence of PWID and injecting drug use in prisons

This section aims to illustrate the willingness of prison staff to speak about injecting drug use in prison and the way they negotiate it. The presented data cannot be used as quantitative evidence of the exact number or percent of PWID in prison, however, it may reflect the prison staff admission of injecting drug use(rs) as being part of the prison system’s day-to-day life and functioning. It was easier for the informants to make estimations in percentages, thus we chose to maintain their way of expression.

The prison staff members’ perception of how many inmates had been injecting drugs before getting imprisoned varied based on the differences between the types of prison facilities (male/female prisons, first time sentenced/several times sentenced). According to the prison staff perception, PWID’s prevalence was estimated at rates that varied from 40% in CC up to 50% in male prisons for men sentenced to imprisonment several times and 100% in female prison for those sentenced to imprisonment several times. The informants’ perception of how many inmates continue to inject drugs while imprisoned ranged between 90% of PWID who continue injecting in male facilities of medium security for those sentenced to imprisonment several times, to 40% continuing injecting drugs in CCs, and minimum or complete absence of any drug use in female prison facilities, where, according to the staff, "*administration fully controls the behavior of prisoners*" (guard, female prison, sentenced to imprisonment more than once).

Respondent 2: You need to understand that in Vasyl Ivanovych’s colony, they get “newcomers,” first-time convicts. And it is 60 [who continue injecting] vs. 40 [who stop injecting] there. While at Borys Petrovych’s it’s 90 vs. 10.

Respondent 1: Right.

Interviewer: 90 vs. 10?!

R2: Yes.

R1: Yes. 90% keep injecting.

I: The repeat offenders?

R1: Yes.

I: And we have heard about SIZOs, that there are many users there.

R2: Yes, many. But the thing is, we do not keep statistics of it, we do not have a narcologist.

(healthcare workers, male prison, first-time sentenced (R1), pre-trial detention center (R2))

I: How many of these people do you think had been using drugs before getting to your institution?

R: About 40%.

I: And how many somehow manage to continue using?

R: The same percentage, yes.

(guard, CC)

I: And among female convicts, did many of them inject drugs before?

R: Drugs? Well, probably, all of them did.

(healthcare worker, female prison, sentenced to imprisonment more than once)

In some cases, the prison staff members’ assessment of PWID prevalence in prisons can be exaggerated and aimed at demonstrating their negative attitudes in regard to certain types of prisoners. This can be especially relevant in the case of female prison staff members who indicated that “probably all” of repeat incarceration women prisoners were injecting drugs before imprisonment. At the same time, according to the interviews with women who were imprisoned, it is likely that being in prison they may indeed have less access to drug use because, in general, they are less likely to retain outside prison connections that are necessary for this. However, according to the informants, women may have access to drug use in other parts of their incarceration experience where the opportunities to socialize with other prisoners who have better outside connections, and thus get drugs, are better—in the pre-trial detention center, on their way to prison, or in the prison hospital. While the prison staff’s duty to oversee and control prisoner behavior constantly lets them track how prisoners act even being outside the facility of their main detention.

There is information within our own channels when the convict, for example, is hospitalized, Me myself, or, well, not me… In short, we learn information about what she is doing there. For example, she smokes pot there. Or eats amphetamines, or swallows something. Well, in general, they have different delicacies there. (guard, female prison, sentenced to imprisonment more than once).

In the CCs, two streams of convicts may cross, some get there for minor offenses, others—as a result of sentence mitigation. According to informants, as a result, "newcomers" are educated by more experienced convicts – people who have committed serious crimes or not serious ones, but on several occasions. This may, in particular, lead to the "newcomers" initiating drug use for the first time while serving their sentence in CC. The informants’ perception of the number of those who start to inject drugs in prison reflected that no more than 10% of people who never injected drugs before start doing it while imprisoned.

R: They [one stream] were transferred here by a court decision – for good behavior, for conscientious work. And, of course, those sitting in the pre-trial detention center [another stream], they came to us for their further stay. That is, here we have a motley audience: starting from the "deadbeat dads" who have not been previously convicted and ending with repeat offenders who have spent their entire adult life in prisons. Such a "hodgepodge". I think from the point of view of the law this is already wrong. Well, how is it possible—a person who has not been previously convicted, has not previously served a sentence in prison, encounters people who have spent their entire lives here.

I: By the way, these people who have spent their whole lives in prisons, are many of them using drugs and, if so, can they get to CC?

R: Yes.

(guard, CC)

The above quote illustrates how prison staff problematize the “newcomers” interaction with repeat offenders. It also illustrates that drug use and criminal acts are perceived as similar forms of behavior, or even as the same behavior, also defined as criminal. At the same time, the above quote illustrates a collective, communal way of living in prisons. Although there may be different “collectives” in prisons, all of them have to learn how to share the space despite possible differences in their backgrounds and (criminal) experiences. In this sense, drug use can be described as one of the few available forms of shared experience between strangers who have to live together. At the same time, the expertise in drug use among prisoners demonstrated by prison staff tells us that surveillance and control of prisoner behavior is carried out constantly.

### Regulating (denial of) access to sterile injecting equipment

Although the activities of a huge number of prison staff are aimed at preventing drugs from getting in and spreading within prisons, it is impossible to track and stop all such attempts. One of the most widespread ways of drug delivery in prison facilities is “tossing” it over the prison wall. However, a prison guard pointed out that according to internal statistics, there is “*1 syringe for 15 drug tosses*” (guard, male prison, sentenced to imprisonment more than once)*.* To seize the prohibited items which were not seized at the “entrance” to prison facilities, raids are systematically carried out by prison guards in the cells and barracks where prisoners live. Thus, if some prisoners have their own syringe for reusable use, then seizing it during a raid leads to the need to use another one that has normally also been used more than once.

We must seize it. If the item is prohibited, it creates its shortage, right? Consequently, prisoners hide syringes. Talking about my personal experience, I have smashed those syringes with my boots during raids more than once because these are prohibited things. (guard, male prison, sentenced to imprisonment more than once)

While the paradox of this situation lies in the fact that in every prison facility where a medical unit is located, needles and syringes are available, “*you just limit their number and make access to them as weakened as possible” (international stakeholder).* As a result, prison health care workers, prisoners who work (help) in medical units,^2^ and ordinary prisoners have to be involved in the informal circulation of the prison’s needles and syringes.

I: How much did the syringe cost, roughly?

R: Oh, then the syringe cost me 40 hryvnias,^3^ one syringe for 5 cubes.

I: Did you top up someone's account?

R: Yes, yes.

I: Someone in the medical unit?

R: Yes, and a new syringe was already coming to me through the medical unit. Well, they said that it was through the medical unit. But again, it's not realistic to check. It's like everything there… Everything happens online. It's not that you come to the medical unit and give cash to someone… The nurse will not take the cash anyway. Of course, if you are in a very good relationship, then she can take it. But basically, everything is done online. Not to be seen, to stay anonymous. They didn't see you, you didn't see them. And at night the prison opens. Because prison always lives at night. And at night you can get a syringe, drugs, and everything.

(ex-prisoner, male prison, first-time sentenced, 38)

It’s not surprising that all this creates harsh conditions for the immediate process of injecting drug use in prisons. The majority of informants referred to their experience of injection equipment reuse until “*marks on the syringe are no longer visible*” (ex-prisoner, pre-trial detention center, 39). At the same time, there is a small group of prisoners who can keep needles and syringes while being in prison, but not in pre-detention centers.^4^ There are exceptions in which a prisoner can legally receive syringes and needles in a medical transfer either in their hands or to use it under health care worker supervision at the medical unit. To do this, a prison doctor should write an appropriate prescription. Such prescriptions can be made in case of treatment that can only be injected. At the same time, this rule does not work in pre-detention centers where a prisoner can get the prescribed treatment through injection only under health care worker supervision at the medical unit (for example in case of diabetes). In general, it is difficult to get access to medical units based at pre-detention centers because to get there the prisoner should be escorted by the guard(s) who are busy with surveillance in the operational environment. Surveillance in pre-detention centers is a hard task—in one such center, there were 2500 prisoners at the time when the study was conducted. The next quote was extracted from an interview with an ex-prisoner who served all his time at the pre-detention center while working (helping) at the medical unit.

My granny brought me syringes, but they didn’t give them to me to keep, they themselves injected me with ceftriaxone [at the medical unit] …. and I had to buy a chocolate or something, a 30 ruble chocolate bar for 1 syringe. And with this one syringe, I was afraid of getting infected with something. I tried to keep it with me at all times. And there were raids, they found the syringe, and it was removed. Because syringes are banned. (ex-prisoner, pre-trial detention center, 32).

A lack of access to syringes, in general, can lead to situations when available syringes will be used by everyone who needs them, not just by the prisoner who was prescribed one to make injections.

R: Yes, he officially had an insulin syringe. The administration knew that.

I: But he used that syringe for six months, you said?

R: Well, not only him. There are periods when you can't get a syringe, buy it at the hospital, or buy it at night, for example. But you have drugs and you want to use it, and you know that there is a person who has a syringe.

(ex-prisoner, male prison, sentenced to imprisonment more than once, 41)

With the help of the Rules underpinned by a perception of drug use itself as a form of criminal behavior, the prison staff members differentiate between two groups of prisoners in need of needles and syringes. The first group is represented by comparatively rare cases of those prisoners who need them and according to the Rules can use needles and syringes for treatment purposes. The second one is represented by the group of “drug addicts” who need needles and syringes “for their own purposes” which are strictly prohibited by the Rules. Since both groups of prisoners live in communal prison conditions and lack access to needles and syringes, both of them may be forced to use one another’s injecting equipment.

### Feasibility of PNSP

Commitment of prison staff to performing their duties in compliance with the Rules was explained as one of the reasons against PNSP feasibility and at the same time an opportunity to implement it in the event that necessary amendments were to be made. Preparing for fieldwork in the frame of this study, we were informed of “an experiment” aimed at condom provision in prisons. It was called an “experiment” because the provision of condoms was not listed anywhere in prison regulation documents while it has been implemented through a separate Order. Determining this experience as a possible example of how PNSP can be implemented, we have been asking the prison staff about condom availability in prisons. Every informant confirmed that condoms are available in prisons, but whether inmates have access to and use them was difficult to answer. For example, the question of whether the condoms are available in the room for long-term visits stunned informants until it finally emerged that condoms are kept by health care workers or other prison staff, so a prisoner should ask in advance of a visit to be given a condom.

I: Tell me, please, is it possible for a prisoner to take condoms? For example, if her husband comes, they can have sex …

R: The medical unit provides it. There's a lot of that.

I: In the room [for long-term visits]?

R: No, the inspector keeps it. Condoms lying around the rooms? It's ugly.

I: Maybe they're in a box.

R: No. The inspector keeps it, if necessary – please ask. We have enough of that.

(guard, female prison, sentenced to imprisonment more than once).

Thus, the availability of condoms was formally ensured through the Order, while neither the algorithm on how to provide them to prisoners was prescribed, nor the need for the confidential provision of this service was considered. In the “ordinary environment,” prescribing algorithms for such service delivery may increase the risk of over-bureaucratization and control, and thus even greater distancing of the "client" from the "service." However, the prison staff commitment to performing their duties in compliance with the order and perhaps their resistance to implementing personally unacceptable interventions suggests that for any intervention to be feasible for implementation step-by-step guidelines should be outlined. Thus, it is probably not a question of “why” a certain intervention is needed but how exactly it should be performed in compliance with the Order. The next quote was retrieved from an interview with a social worker who used to work at SPS previously.

I left the penitentiary service and went to work at a school. When you work in a power structure you work according to the principle that you have orders. That is difficult to readjust when you work like that, to work without instruction. Let's say, two years have been a process of adaptation. It was difficult to rebuild psychologically. Because when you are a boss it is one thing, and then when you are an ordinary person it is another. (social worker, male prison, sentenced to imprisonment more than once).

At the same time, the pervasive power of constant surveillance and internal control as a main prison staff member’s duty seems a key problem for any external intervention and systemic changes. The same way the Rules prescribe prison guards to violate prisoners’ personal space, the practice of its everyday application violates the borders of their own life not leaving room for their other possible roles. In the interviews with prison guards, for example, their duty to control as a professional duty often bordered with their special personal commitment “to live like a guard.” Although some discussed this role as natural, others compared it with the sentence they have to serve.

A guard is such a person's essence. You have to be a guard [to understand]. To a certain extent, I have a distrust of doctors, of those, of those. You see, I distrust 90% of those with whom I work. This is my job, I am a guard. I suspect anyone. (guard, pre-trial detention center)

R: No, it does not have a future.

I: You mean the penitentiary service?

R: Yes, I mean the penitentiary service. Why? Because before, when there was an institution, there were still old laws, there was a law that allowed us the right to work for 13 years. That Law recognized that the person is already irreversibly deformed after 13 years. The influence that SPS had on him no longer passes. But now for some reason, they said 25 years. Well, 25 years is a life sentence.

And: Well, you have the right to leave this place.

R: No, you are wrong. Some services, structures, and so on—yes, but we do not. For example, I have had 1.5 days off in the last 10 days.

(guard, male prison, sentenced to imprisonment more than once)

The feasibility of PNSP was rarely discussed by prison staff members in terms of the intervention aims of reducing harm to prisoner health. Mainly, it was discussed as a question of the frightening possibility of the loss of control over prisoners and the legitimacy of a different order than the present one.

They will take them, and will exchange, and they will demand to exchange them. And demand and demand it. But if this happens, well, I don't know why then our structure will be needed. God knows what will happen here. Much worse than it is. <  > It seems to me that it is better to give a person a gun. For fifteen years we will shoot each other, and then we will understand [how to use it], we will respect each other. (guard, male prison, sentenced to imprisonment more than once).

I think this system is so closed. Everything runs in a loop here. One boss replaces another boss, and often when I personally come to some penitentiary institution, I find things that even they do not pay attention to. For example, there is a wall stand hanging there and the 1960 Criminal Code is presented there. That is, those people from the system, they can just pay attention to the fact that people who have been in the system for years, they just forget about it and it does not matter to them. For them, the essence of punishment is to condemn a person, to punish him, to keep him in harsh conditions, and not to give him any rights. And that's why they don't think about it, they don't understand it at all. (SPS regional head).

The informants stressed the role of so-called “political will” being essential for further SPS development and would only make PNSP implementation conceivable. Among other “pills” for change is “moving away from the Soviet past” which was consistently mentioned in the interviews. This need for systemic changes was voiced by informants occupying different positions within SPS. Such voices at the level of ordinary prison guards along with regional heads have been encouraging.

It is trendy to call it “political will.” For me, it is a question of responsibility for making managerial decisions, that's all. A person occupies a certain position, if he is responsible for HIV prevention, he must develop these policies, he must take the most effective international practice, and act accordingly. What is “access to syringes?” It is an intervention that interrupts the chain of HIV infection. That is all—access to sterile instruments is needed. (national stakeholder).

I would like to stay an employee in this service, there are prospects, and I want it to become better. And so I want us to move away from the Soviet space, rules, and regulations as a custom, but not as in Europe. (guard, male medium).

To summarize, the PNSP can be considered as a simple and effective measure to reduce injecting drug use harm, while in the discursive universe of prison staff a special and complex meaning can end up being assigned to this intervention. In fact, not to the intervention itself, but to the changes that can happen as a result of its implementation—even leading to the collapse of the prison order. At the same time, other informants believe that systemic changes and political will are needed first in order to implement PNSP.

## Discussion

The prison staff members’ estimations on how many inmates had been injecting drugs before getting imprisoned and continue to do it while imprisoned is consistent with quantitative studies conducted in Ukraine and in other countries in the region [17, 55, 64, 65]. According to the studies conducted in Ukraine in 2015–2019, between 30 and 47% of the prison population have experienced injecting drug use before imprisonment [17, 55, 64]. According to the Integrated Bio-Behavioral Survey conducted among prisoners in Ukraine in 2019, 41% of those prisoners who replied to the question on other inmates' drug use, pointed out that other prisoners inject opiates while imprisoned [64]. According to various studies undertaken in Europe, between 16 and 60% of people who injected before their incarceration continue to inject in prison [17]. A study conducted in post-Soviet Kazakhstan indicated that 85% of prisoners with a history of injecting drugs continue to inject drugs while in prison [65]. It is noteworthy that normally such estimates are made through surveys conducted among current prisoners or among those who were recently released, while the presented study offers an opportunity to learn what estimations can be made by the prison staff members and how these estimations are negotiated as a form of control over the prisoners’ behavior and situation. We argue that the presented findings are useful for making PNSP feasible in Ukrainian prisons in terms of being “evidence” that injecting drug use takes place in prisons. This evidence, according to the WHO, justifies prison authorities starting PNPS.

At the same time, based on the opinions of informants, we argue that in the researched field there might not be enough evidence to start PNSP. Thus, the presented findings can also be considered as a broader reflection of the political and cultural environment in Ukraine. On the one hand, the interviewed prison staff admit to the existence of injecting drug use in prison. This can be considered as a sign of prison staff openness, which appeared as a result of prison reform started in 2016, the post-Revolution effect of liberalization, and an overall change of the social-political direction towards “western values” [66–68]. On the other hand, this “openness” can only partly be a result of the described processes, while another reason is that the discussed problems are not of an extraordinary nature and more of a routine for the prison staff. This may sound like a big assumption, but if Ukraine is stuck in the continuing cycles of revolution, disillusionment and stagnation since 1991 [52], the punitive drug policies remain punitive as a tradition, which developed from earlier Soviet times [43, 69], and forces PWID to regularly go through the cycles of imprisonment-release-imprisonment [70]. The systematic nature of these cycles makes PWID prevalence in prisons commonplace, and this makes PNSP implementation genuinely relevant.

The prevalence of injecting drug use in prison as a collective and socializing practice is embedded remarkably in the logic of post-Soviet prison infrastructure, in organizations and functioning where Soviet legacies are still dominant. The presented findings can be considered as an illustration of how Soviet legacies continue to work in the post-Soviet prison environment. The Makarenko pedagogy is represented here through its other side—prison collectives may not only produce and reproduce criminal subculture but, using the same mechanisms, produce and reproduce drug use in prison as well. This may also partly explain the “prosaic” intonations of prison staff while discussing drug use in prisons and their grounds since the criminal subculture being the alternative governance body apart from the prison administration as part of the Soviet penitentiary system is an old and well-studied phenomenon [38, 39, 71–74]. Thus, the process of the creation of collectives and their resilience in prison is not new for the prison staff. At the same time, it is obvious that this enforced carceral collectivism that results from the communal and poor conditions of the enclosed environment produces the practice of sharing, including needles and syringes as an inevitable part of such shared living. In one of the studies of post-Soviet Kyrgyz prisons [75, 76], by referring to post-Soviet collective identities as the opposite of the individualism of the West, the authors suggest “that a healthy body is produced through a healing process that rests on submission to the collective, with a constant threat of exile. In such settings, health care interventions aimed at the individual are unlikely to succeed without consideration of collective healing practices” [76]. It could be suggested, that the mentioned observation should be considered in regard to any intervention in the process of integration into the post-Soviet prison system. Understanding how the legacies of communal and collectivized living are embedded in the lives of incarcerated people is particularly relevant for PNSP implementation.

According to this study’s findings, the number of needles and syringes circulated in prisons is low, while the prison guard raids reduce their number to a minimum. This seems to be a peculiar mixture of the prison guard power over prisoners and a way that punishment for prisoner drug use is implemented at the same time. This finding also illustrates the specific nature of this “power over” [38]. The prevalence of injecting drug use and admission among the prison staff of this fact demonstrates that the drugs are not under the same control as the prisoners and their behavior which is controlled through regulating their access to drugs and injection equipment. Implementation of PNSP could remove the basis of this power and change the ways the prisoners and staff interact. However, any model of PNSP should be analyzed for any unanticipated harm that could result from its poor planning and regulatory practices [77–80]. The described case of condom distribution can be considered as an example to anticipate unintended consequences of PNSP implementation in Ukrainian prisons. However, this possibility needs elaboration through further research.

The “political will” mentioned consistently as a key feature in implementing any intervention in prisons, including systemic changes, can be explained through its double meaning in the studied context. Although it should save the prison system from destruction caused by its outdated Soviet order, it would be itself an embodiment of a prison system order—a power vertical over the prison staff themselves. At the same time, it may also be true that without such a will incorporated into the Rules any changes are impossible in the current environment. Furthermore, it may be useful to consider political will as an intervention aimed at horizontal collective changes of prison staff, and not only as imposing the will of the SPS authorities.

## Conclusion

Thus, it can be concluded that PNSP implementation is not just a question of having evidence of injecting drug use in the hands of prison authorities. It also relies on the political climate and ideology of a particular time and historical period significantly reflecting the feasibility of PNSP. Or, in other words, for PNSP to be feasible in the prison environment, there is a need for specific changes to transition from one historical period and political leadership to another. And, thus, to make PNSP work requires making the power work for change and not just for reproducing the power itself. This issue is of particular relevance taking into account the responsibilities of the Ukrainian Government to fulfill its international obligations under the International Covenant on Economic, Social and Cultural Rights in light of the recent recommendations of this treaty’s monitoring body to expand harm reduction to prisons.

## Data Availability

Please contact the Public Health Center of the Ministry of Health of Ukraine for data requests. The data belongs to the Public Health Center of the Ministry of Ukraine and needs permission to be provided.

## Abbreviations

WHO: World Health Organization
PWID: People Who Inject Drugs
PNSP: Prison Needle and Syringe Programs
SPS: State Penitentiary Service
OAT: Opioid Agonist Therapy
PAR: Participatory Action Research
MOJ: Ministry of Justice
CC: Correction Centers

## Footnotes

1 SIZOs = pre-trial detention centers [abbreviation from Ukrainian/Russian].

2 Such prisoners are also known as “activists” or “trustees,” they are occupied with soft jobs (in medical units or the prison kitchen) helping out the prison staff members.

3 40 hryvnias = 1.5 USD, this price is approximately 10 times higher than in the public sector.

4 In 2019, this rule was changed—now, according to the list of prohibited items presented in the Rules, prisoners have the right to keep “5 sealed needles and syringes necessary to maintain the vital functions of the body in the presence of an appropriate medical prescription” in pre-detention centers.
